# Case Series: Pulmonary Artery Intramural Hematoma in Stanford Type A Acute Aortic Dissection

**DOI:** 10.5334/jbsr.2446

**Published:** 2021-06-08

**Authors:** Jeanne Gros-Gean, Olivier Lebecque, Alain Nchimi, Mihaela-Magdalena Vlad

**Affiliations:** 1 Université catholique de Louvain, CHU UCL Namur, Department of Radiology, 1 Avenue Dr G Thérasse, 5530, Yvoir, BE; 2 Centre Hospitalier du Luxembourg, CHL Luxembourg, 4 Rue Nicolas Ernest Barblé, 1210, Luxembourg, LU

**Keywords:** Stanford type A acute aortic dissection, pulmonary artery intramural hematoma, pulmonary embolism, alveolar hemorrhage, aortic wall rupture, fluoroquinolones

## Abstract

**Main Teaching Point:** Diagnosing acute ascending aortic dissection in patients with equivocal radiologic data may rely on associated findings such as pulmonary artery intramural hematoma.

The immediate diagnosis of aortic dissection is paramount in its management. Its diagnosis may be challenging on computed tomography when the intimal flap, pathognomonic of dissection, is not readily visualized. Pulmonary artery intramural hematoma may arise from rupture of the posterior wall of the ascending aorta into the common aortopulmonary adventitia as a result of acute dissection. The clinical significance of pulmonary artery hematoma is unknown, but its presence may facilitate the diagnosis of acute dissection when other radiologic findings are equivocal. Herein, we present four cases of pulmonary artery intramural hematoma associated with Stanford type A acute aortic dissection, among whom patient outcomes depended mainly on the prompt treatment the dissection.

## Introduction

Pulmonary artery intramural hematoma (PA-IMH) is a rare complication of acute aortic dissection (AAD) [1]. When associated with Stanford type A AAD, PA-IMH may result from blood extravasation secondary to rupture of the posterior wall of the ascending aorta into the common aortopulmonary adventitia [1]. PA-IMH may be detected on computed tomography (CT) as a hyper-attenuated crescentic or circumferential thickening of the pulmonary arterial wall [1].

On the other hand, the aortic intimal flap, a pathognomonic feature of aortic dissection, may not be readily visualized on imaging, thereby posing a diagnostic challenge. In such cases, PA-IMH may be a sign of AAD. The clinical significance of PA-IMH is unknown so far. Herein, we present four cases of PA-IMH associated with Stanford type A AAD with various clinical and radiologic presentations in which clinical outcomes mainly depended on the diagnosis and management of AAD.

## Case 1

A 61-year-old woman with a history of bilateral lung transplantation, left upper lobectomy, arterial hypertension, and diabetes presented to the emergency department with chest pain of sudden onset. She was hypertensive (blood pressure, 186/89 mmHg) and had an elevated troponin level of 0.55 ng/mL (normal: <0.12 ng/mL). Serum creatine kinase and D-dimer levels were normal.

An electrocardiogram (ECG) showed negative T-waves and ST depression, which likely suggested non-ST segment elevation myocardial infarction. Transthoracic echocardiography (TTE) revealed ascending aortic dilatation, aortic regurgitation, and AAD. Although the pulmonary artery was dilated, the right cardiac chambers were normal. Pulmonary pressures were not evaluated.

**Figure 1:**
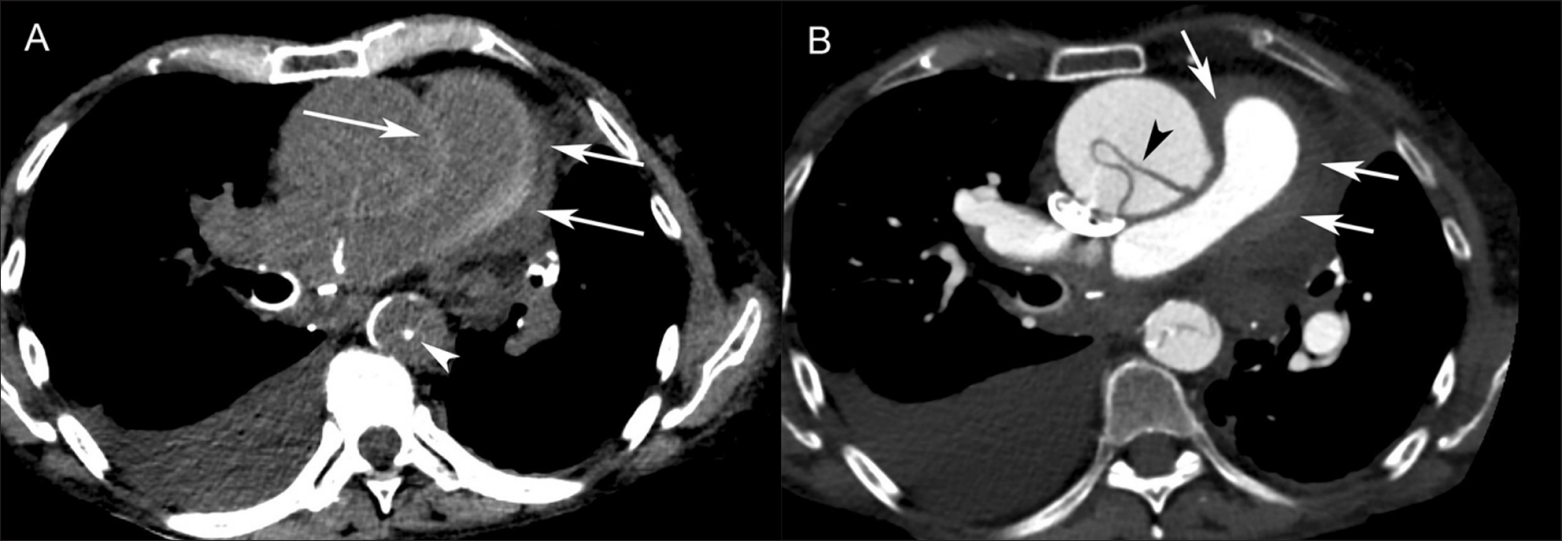
Patient 1. (**A**) Axial non-contrast-enhanced CT showed circumferential high attenuation along the walls of the pulmonary trunk (70 HU) and right pulmonary artery (arrows), consistent with an intramural hematoma. An ascending aortic aneurysm with displacement of atherosclerotic calcifications into the lumen of the descending aorta (arrowhead) was seen. Right pleural effusion was also visible. (**B**) Contrast-enhanced CT revealed Stanford type A aortic dissection. The intimal flap was seen in the ascending aorta (arrowhead) and descending aorta. The high attenuation area of the pulmonary arterial wall was non-contrast-enhancing (arrows), which was consistent with pulmonary artery intramural hematoma.

An ECG-gated contrast-enhanced CT angiography (CTA) scan revealed Stanford type A dissection involving the aortic root, extending into the descending aorta and an ascending aortic aneurysm (maximum diameter, 5.4 cm). On the pre-contrast CT scan, there was a circumferential high attenuation (70 HU) along the walls of both the pulmonary trunk and arteries with mild luminal narrowing (***Figure 1***). The thickened wall of the pulmonary arteries was not enhanced with contrast, indicating an intramural hematoma (***Figure 1B***). Alveolar hemorrhage was not observed. The patient underwent ascending aortic replacement. Two weeks postoperatively, she developed a paramediastinal hematoma, which slowly regressed after one month.

## Case 2

**Figure 2:**
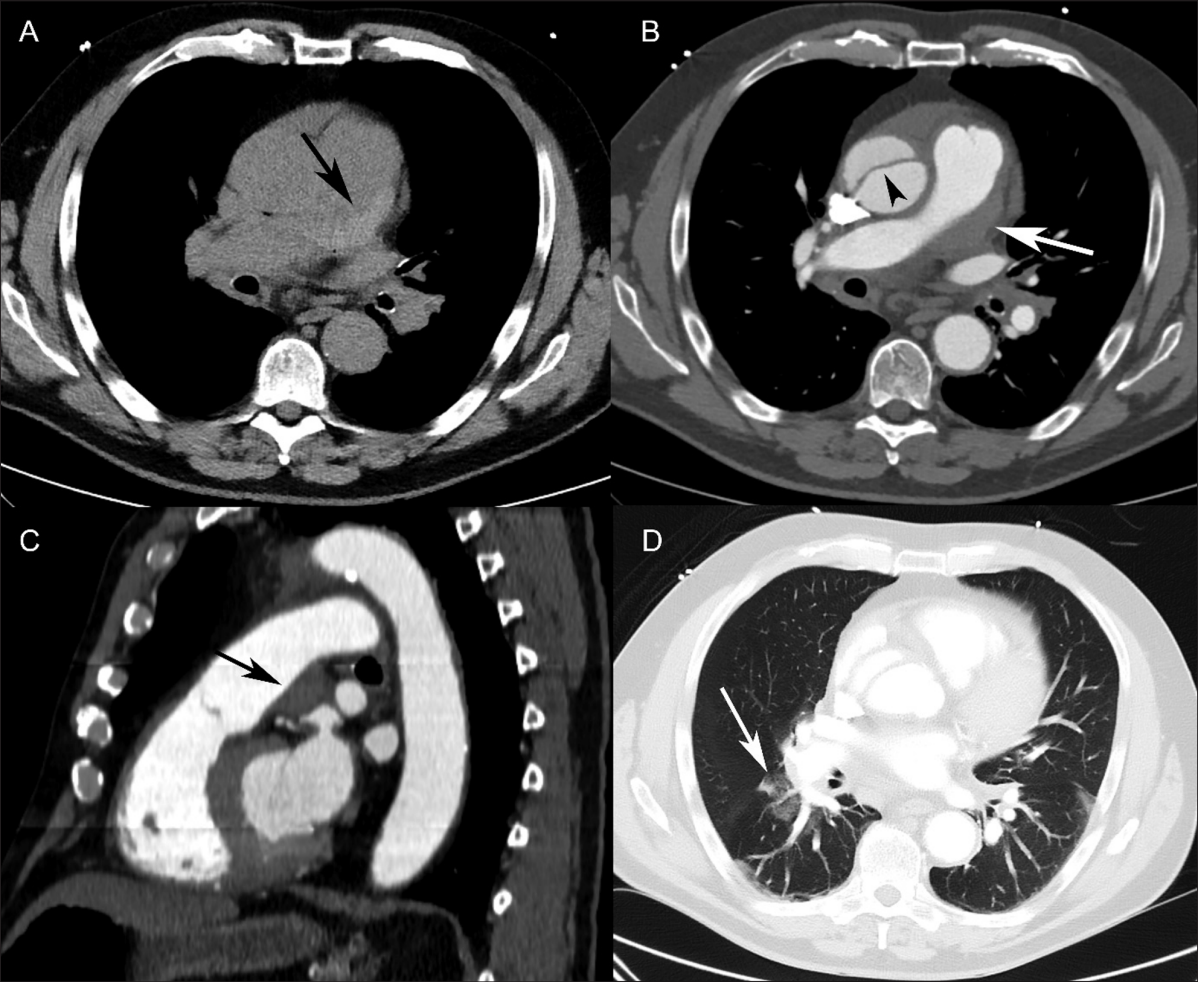
Patient 2. (**A**) Axial non-contrast-enhanced CT showed a hyperdense lesion along the pulmonary trunk wall (black arrow). (**B**) Contrast-enhanced CT (axial view) revealed an eccentric hypodense defect in the pulmonary trunk (white arrow), mimicking a mural thrombus. The intimal flap was seen in the ascending aorta (arrowhead). (**C**) Sagittal reformatting of a contrast-enhanced CT scan revealed a lesion with well-defined margins at an obtuse angle (black arrow) along the pulmonary trunk wall, resulting in a slight narrowing of the pulmonary trunk lumen. This was not enhancing, suggestive of an intramural hematoma. (**D**) Axial CT (lung window) showed patchy ground-glass opacities in the right lower lobe around the segmental pulmonary arteries, consistent with alveolar hemorrhage.

A 67-year-old man with a history of smoking and obesity was referred to our emergency room on account of chest pain of sudden onset after loading water packs into his car. TTE revealed aortic bulb dilatation, minimal pericardial effusion, and an intimal flap in the ascending aorta. Non-contrast-enhanced CT revealed a crescentic high attenuation of the pulmonary trunk wall (***Figure 2A***). CTA revealed a Stanford type A aortic dissection with an intimal flap in the ascending aorta (***Figure 2B***). The area of high attenuation along the wall of the pulmonary trunk was not enhanced with contrast, suggestive of an intramural hematoma (***Figure 2B*** and ***2C***). The pulmonary artery (maximum diameter, 3.5 cm) and right cardiac chambers were dilated on CT. There were patchy ground-glass opacities in the right inferior lobe around the segmental pulmonary arteries, indicating alveolar hemorrhage (***Figure 2D***). Throughout the examination, the pain persisted with associated signs of shock and cardiac tamponade. Prompt resuscitative maneuvers did not restore the hemodynamics, and the patient died.

## Case 3

**Figure 3:**
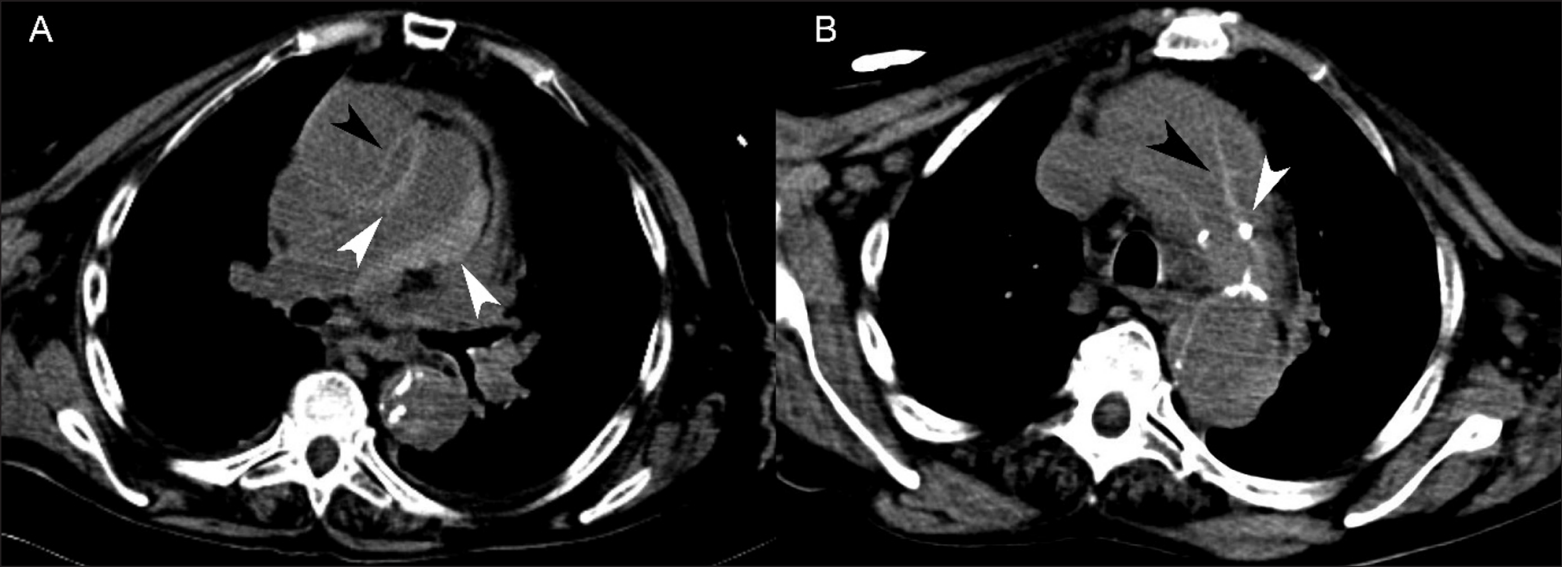
Patient 3. (**A**) Non-contrast-enhanced CT showed circumferential high attenuation along the walls of the main and right pulmonary arteries, consistent with pulmonary artery intramural hematoma (white arrowheads). Stanford type A aortic dissection with an intimal flap in the ascending aorta was also seen (black arrowhead). (**B**) Non-contrast-enhanced CT revealed an intimal flap of the thoracic aorta (black arrowhead) and displacement of atherosclerotic calcifications into the aortic lumen (white arrowhead).

A 91-year-old woman with a history of arterial hypertension and congestive heart failure was found unconscious on the floor, which prompted her transfer to the emergency department. The use of contrast was contraindicated due to renal failure with elevated creatinine levels of 1.68 mg/dl (normal: 0.52–1.04 mg/dl). Non-contrast-enhanced CT revealed an intimal flap with displacement of atherosclerotic calcifications into the lumen of both the ascending and descending aortas, consistent with a Stanford type A aortic dissection (***Figure 3A*** and ***3B***). A circumferential area of high attenuation along the wall of the main and right pulmonary arteries was also observed, suggestive of PA-IMH (***Figure 3A***). Alveolar hemorrhage was not observed. The patient died shortly after.

## Case 4

**Figure 4:**
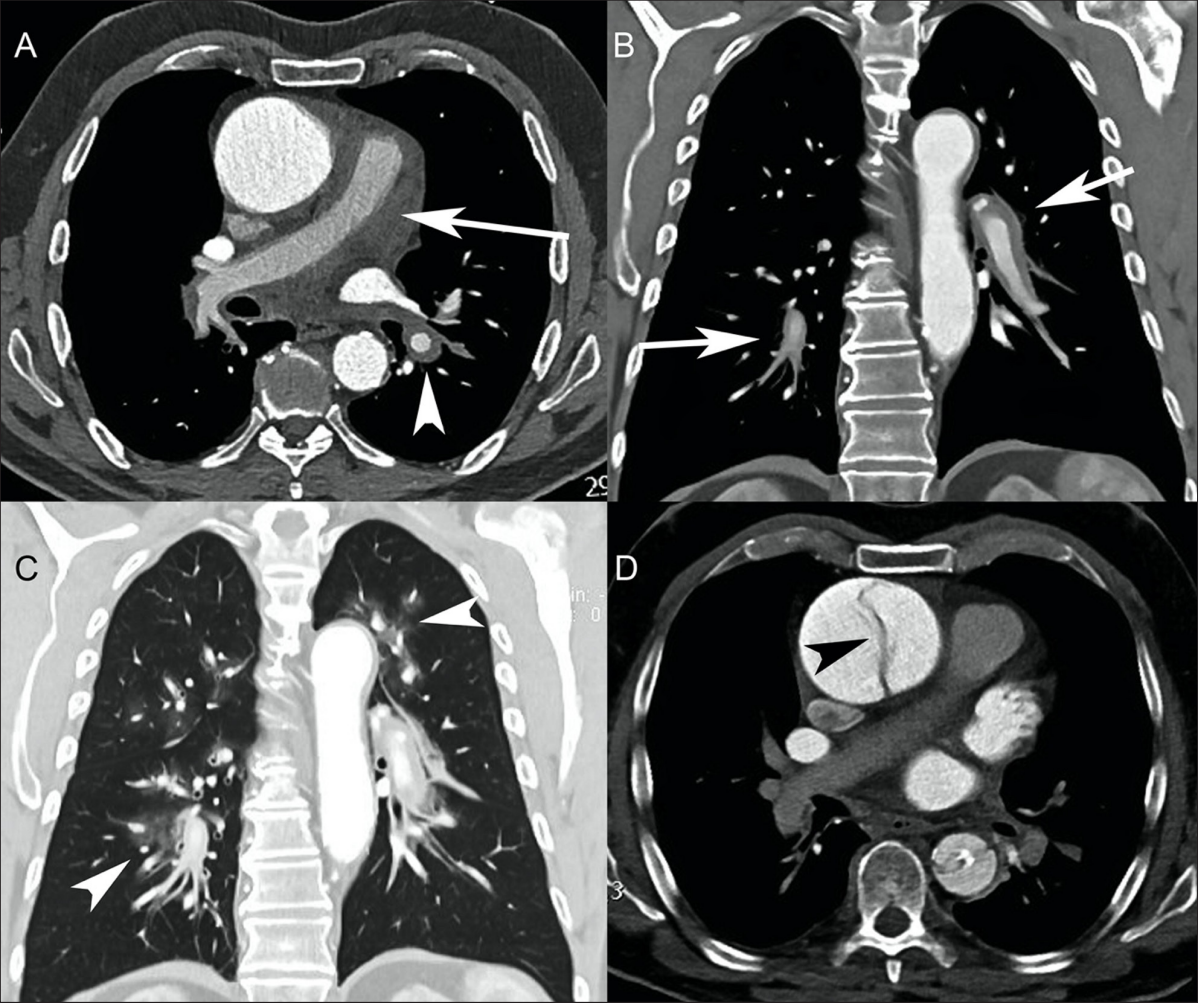
Patient 4. (**A**) Initial axial CT angiography (CTA) showed an aortic aneurysm. No intimal flap was seen. However, the contour of the ascending aorta was irregular. There was circumferential thickening of the pulmonary arterial wall (arrow), extending into the right and left pulmonary arteries (arrowhead), suggestive of pulmonary artery intramural hematoma. (**B**) Initial CTA (coronal reformation) showed the extension of the pulmonary artery intramural hematoma to the interlobar and segmental pulmonary arteries (arrows). (**C**) Initial CTA (coronal reformation, pulmonary window) revealed ground-glass opacities around the pulmonary arteries consistent with alveolar hemorrhage (arrowheads). (**D**) Second axial CTA performed two days later showed an intimal flap of the ascending aorta, suggestive of Stanford type A acute aortic dissection (arrowhead). Both the pulmonary artery intramural hematoma and alveolar hemorrhage had resolved.

A 69-year-old man with a history of Parkinson’s disease, ascending aortic aneurysm, and bilateral hip replacement was transferred to the emergency department from another institution due to suspected acute aortic syndrome. CTA performed before the transfer was equivocal for dissection and showed irregular contours in the ascending aortic aneurysm. The pulmonary arteries were thickened, suggestive of PA-IMH (***Figure 4A*** and ***4B***). Central ground-glass opacities were also seen, consistent with alveolar hemorrhage (***Figure 4C***). On admission, the patient was taking three classes of antibiotics (including a fluoroquinolone) due to a methicillin-sensitive *Staphylococcus aureus* infection following hip replacement. Physical examination revealed a systolic murmur in the aortic region. TTE revealed severe aortic regurgitation, non-compressive pericardial effusion, and normal pulmonary flow velocity gradient. Thirty-six hours later, a repeat CTA scan unequivocally revealed a Stanford type A aortic dissection (***Figure 4D***). Interestingly, by the time of the second CT scan, both the PA-IMH and alveolar hemorrhage had disappeared. The patient underwent an emergency Bentall procedure, had an uneventful recovery, and was discharged two weeks later.

## Discussion

Acute aortic syndrome encompasses intramural aortic hematoma, penetrating atherosclerotic ulcers, and AAD, which are life-threatening aortic emergencies [2]. PA-IMH can occur alone or in association with acute aortic syndromes, most frequently Stanford type A AAD [3,4]. PA-IMH is usually considered a rare complication of Stanford type A AAD; however, studies have shown that 9–16% of patients with Stanford type A AAD present with hemorrhage extending along the pulmonary artery [1,5].

Anatomically, the ascending aorta, main pulmonary artery, and pulmonary arteries (left and right) share a common adventitia [6]. Among patients with rupture of the posterior wall of the aortic root, the extravasated blood may enter the common adventitia, easily extending in the adventitial space of the main pulmonary artery and thereafter into the adventitial space of the left and/or right pulmonary arteries, resulting in PA-IMH [4,6].

The diagnosis of PA-IMH is generally established via non-contrast-enhanced CT, which typically shows hyperdense thickening of the pulmonary arterial wall [1]. PA-IMH may easily be overlooked on contrast-enhanced CTA. Therefore, non-contrast-enhanced imaging should be included in the CT protocol of patients with suspected acute aortic syndromes to improve the detectability of IMH and PA-IMH [5].

In some patients, PA-IMH can compress the pulmonary arterial lumen, leading to pulmonary artery obstruction [7]. Thus, on CTA, PA-IMH may be mistaken for pulmonary embolism, particularly if crescentic in shape [8]. Moreover, PA-IMH is non-enhancing and becomes hypodense compared to the lumen on CTA [5], causing thin PA-IMH to be missed.

In severe cases, the vessel may continue to dissect into more peripheral pulmonary branches and extend into the alveoli, leading to alveolar hemorrhage. This CT finding has been reported to be a significant prognosticator of mortality [1]. However, in our study, alveolar hemorrhage in PA-IMH seemed to resolve quickly and did not influence patient outcomes. The non-futility of rapid and effective surgical treatment is the main determinant of survival among patients with Stanford type A AAD, which was confirmed by the survival of only the patients who underwent surgical treatment (***Table 1***). In the longer term, it is unknown the extent to which PA-IMH would not result in hazardous sequelae such as pulmonary hypertension or aneurysmal dilatation of the pulmonary arteries, due to pulmonary arterial wall damage [9].

**Table 1:** Summary of the radiologic findings and clinical outcomes of the four patients with acute aortic dissection and pulmonary artery intramural hematoma.


	CASE 1	CASE 2	CASE 3	CASE 4

Alveolar hemorrhage	No	Yes	No	Yes

Right ventricular dysfunction	None	Yes	None	None

Surgery	Yes	No	No	Yes

Outcome	Alive	Death	Death	Alive


The most effective preventive measure for AAD is to control modifiable risk factors such as smoking and arterial hypertension. Recently, fluoroquinolones have been implicated in the development of aortic diseases through increased degradation of wall collagen [10]. Fluoroquinolone use doubles the risk of aortic aneurysm or dissection within 60 days of treatment and induces an accelerated increase in aortic diameter [11]. This stresses the importance of regularly assessing patients taking fluoroquinolones for classical risk factors of aortic disease. Despite this, the fourth patient suddenly developed AAS for which the initial CT findings were equivocal. The presence of PA-IMH, however, expedited the diagnosis of AAD.

Lastly, other possible complications of AAD may aid in arriving at a differential diagnosis when radiologic findings are equivocal. These include hemopericardium, hemomediastinum, and hemothorax, which occur when blood extravasates into the pericardial sac, mediastinum, and pleural space, respectively [1,4,6].

## Conclusion

PA-IMH is a rare complication of Stanford type A AAD following blood extravasation from the ruptured aorta into the common aortopulmonary adventitia. PA-IMH appears on non-contrast-enhanced CT as a hyper-attenuated thickening of the pulmonary arterial wall that is more subtly depicted on CTA since it does not enhance.

The presence of PA-IMH may aid in the diagnosis of AAD when the aortic intimal flap is not visualized. Furthermore, PA-IMH does not greatly influence immediate clinical outcomes, which mainly depend on the prompt diagnosis and surgical treatment of AAD. The long-term sequelae of PA-IMH have not been entirely elucidated.
